# Future land use maps for the Netherlands based on the Dutch One Health Shared Socio-economic Pathways

**DOI:** 10.1038/s41597-024-04059-5

**Published:** 2024-11-16

**Authors:** Martha Dellar, Gertjan Geerling, Kasper Kok, Peter M. van Bodegom, Gerard van der Schrier, Maarten Schrama, Eline Boelee

**Affiliations:** 1 Institute of Environmental Sciences, University of Leiden, Van Steenis Building, Einsteinweg 2, 2333CC Leiden, The Netherlands; 2 Deltares, Daltonlaan 600, 3584BK Utrecht, The Netherlands; 3 Environmental Systems Analysis, Wageningen University, P.O.Box 47, 6700AA Wageningen, The Netherlands; 4 Royal Netherlands Meteorological Institute, Utrechtseweg 297, 3731GA De Bilt, The Netherlands

**Keywords:** Environmental impact, Socioeconomic scenarios, Environmental health

## Abstract

To enable detailed study of a wide variety of future health challenges, we have created future land use maps for the Netherlands for 2050, based on the Dutch One Health Shared Socio-economic Pathways (SSPs). This was done using the DynaCLUE modelling framework. Future land use is based on altitude, soil properties, groundwater, salinity, flood risk, agricultural land price, distance to transport hubs and climate. We also account for anticipated demand for different land use types, historic land use changes and potential spatial restrictions. These land use maps can be used to model many different health risks to people, animals and the environment, such as disease, water quality and pollution. In addition, the Netherlands can serve as an example for other rapidly urbanising deltas where many of the health risks will be similar.

## Background & Summary

The Shared Socio-economic Pathways (SSPs) are global scenarios covering a broad range of environmental and societal factors^1^. They enable researchers and policy makers to consider possible future challenges and to analyse how best to respond to such challenges. While these global SSPs are a valuable resource, they are also very broad, making them difficult to apply at a national or regional level, or to use for detailed study of a specific topic. To allow for the inclusion of local context, policies and customs, the SSPs have been downscaled for several countries, making them a more useful tool for policymakers. Recently, the Dutch One Health SSPs were released^2^, marking the first time SSPs have been created for the Netherlands. This was also the first time SSP scenarios have been created with a focus on One Health, i.e. aimed at sustainably balancing and optimising the closely linked health of people, animals and the environment^3^. While scenarios have been developed to look at specific health risks (e.g. heat stress) for individual countries, they had not previously been designed to take a more general approach to health or to have such broad applicability^4^. Since many health risks are inter-related and have non-additive effects^5^, it is helpful to take this more holistic One Health view when considering possible future health challenges. The Dutch One Health SSPs encompass factors affecting human, animal and environmental health and are designed to be used to analyse many different possible future health risks in the Netherlands, a country which can serve as a model for rapidly urbanising deltas around the world. They encompass SSPs 1, 3, 4 and 5 and describe how the Netherlands might look in 2050.

The Dutch One Health SSPs are predominantly qualitative scenarios. For them to be used to model future health risks, it is necessary to add more quantitative information. The creation of future land use maps is particularly helpful here, since many health risks are related to land use. For example, land use affects the hydrological cycle, with multiple consequences for health, such as drinking water being affected by groundwater quality^6^. Mosquito abundances have been associated with land use, as has the emergence of infectious diseases^7,8^. Land use can also affect air pollution, thus affecting respiratory health, and has also been found to have effects on mental health^9,10^. Land use maps for future scenarios thus enable researchers to quantitatively model specific health risks, informing future planning and preparedness. Of course these scenarios do not cover all possible eventualities, but they nevertheless cover a wide range of possible futures and as such should be a valuable resource. We chose to use the Dutch One Health SSPs as the basis for land use maps to assess future health risks given their focus on factors relating to health, and also because they are broadly in line with other future scenarios for the Netherlands^11,12^, suggesting that these are among the more likely future pathways for the country.

Urban deltas have been identified as particularly vulnerable ecosystems, with the combined pressures of high population density and rising sea levels^13,14^. They face many different health challenges, such as loss of natural habitat, salinisation, flooding and disease introduction^14–16^. Land use maps are a useful tool for understanding these challenges and lessons learned from studies in the Netherlands will be helpful for other urbanised deltas. In addition, the methods and assumptions used here can be applied to studies in other places; we detail all assumptions made and all parameters used to inform similar future work. We have created a land use map for each of the Dutch One Health SSPs for the year 2050 using the DynaCLUE land use model^17^.

## Methods

To create land use maps for each of the Dutch One Health SSPs, we used the DynaCLUE model^17^. This model combines an empirical analysis of location suitability with a dynamic simulation of the competition and interactions between land use types, both spatially and temporally. It allows for autonomous changes over time, spatial restrictions on changes and the influence of the surrounding neighbourhood^18^. The model has four main input types, corresponding to steps two, three, four and six below, to which it gives the following names:

- Land use type specific conversion settings
- Land use requirements (demand)
- Location characteristics
- Spatial policies and restrictions

In the following sections, we explain what these inputs are and how they were determined. DynaCLUE runs as a stand-alone program; all other analysis was performed in R v4.0.4^19^.

### Classify land use types

We distinguish between six land use types: urban, pasture, crops, forest, non-forest nature and water. We decided that additional types beyond these six would add unnecessary uncertainty to our maps and that these were sufficient for determining major trends in future health risks. Indeed, many studies which consider the effects of land use on health use just a few very general land use types, thus increasing their applicability^6,8,20^. Our chosen land use types are based on the CORINE land use types^21^, the full classification scheme is shown in table SM1 in the supplementary materials on Zenodo^22^. We used the 2018 CORINE land cover dataset^23^ to map the current Dutch landscape based on these six types. The CORINE dataset uses a 100 m grid, but we used a 1 km grid for our future maps, since this both increased computational efficiency and was more aligned with the expected prediction power of the land use maps we were making. Each 1 km gridsquare was assigned its most prevalent land use type, which on average accounted for 83% of the gridsquare. This provided the starting land use for our simulations.

### Use historic Dutch land use data to determine the likelihood of changes (Land use type specific conversion settings)

**Fig. 1:**
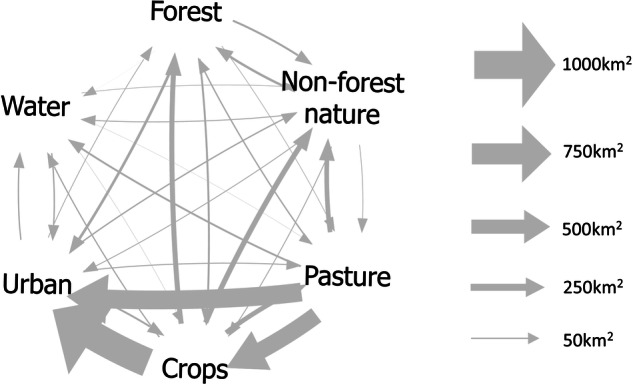
Historic land use changes in the Netherlands from 1990 to 2018, based on a 1 km grid. Arrow thicknesses indicate the land area which changed.

DynaCLUE needs to know what land use conversions are possible, how hard it is for them to happen, and how long they take. To determine these, we used the CORINE land cover datasets for 1990^24^, 2000^25^, 2006^26^, 2012^27^ and 2018^23^ to see what land use changes had previously occurred in the Netherlands and how often. The findings are summarised in Fig. 1.

DynaCLUE requires a ‘conversion elasticity’ for each land use type, indicating how difficult it is for it to change to a different type, regardless of what type it is changing into. These are on a scale from zero to one, where zero indicates that changes are very easy, and one indicates that changes are impossible. We derived conversion elasticities for each land use type by scaling the number of times each land use type had changed in the past. These are shown in Table 1. SSP3 is a low investment scenario where things mostly stay the same. For this reason, we increased the elasticities for pasture and crops for this scenario (to 0.4), making them less likely to change. For all other scenarios the elasticities used were those shown in Table 1.

**Table 1:** Number of changes since 1990 and conversion elasticities for each land use type.

Land use type	Number of changes from this land use type	Conversion elasticity
Urban	234	0.90
Pasture	1630	0.04
Crops	1678	0.01
Forest	270	0.88
Non-forest nature	215	0.91
Water	91	0.99

We decided to remove water areas from the analysis, since it has changed so little in the past and is unlikely to change before 2050. This simplifies the model and increases the likelihood of convergence. While the Dutch One Health SSPs do describe some major changes involving both land to water and water to land conversions as a response to rising sea-levels, these do not occur until the second half of the century.

Length of time between changes is also incorporated into DynaCLUE, e.g. once an area becomes cropland, is there a maximum or minimum time it will stay like that before it becomes an urban area? From the historic data, it seemed that almost all changes were possible and most happened at least once within relatively short time periods. While this might seem a little unrealistic, we decided not to add additional restrictions. It seems possible that it is due to the scaling to a 1 km grid. Also, our analysis is largely based on the CORINE datasets, which include these fast changes, and it seemed inadvisable to try to model dynamics which were not present in the datasets we were taking to be ‘true’.

### Determine demand for each land use type under each scenario (‘Land use requirements (demand)’)

DynaCLUE requires a ‘demand’ for each land use type for each year simulated. The number of gridsquares of each type for the current situation provides the starting demand for modelling future land use change. The demand for the future scenarios must be determined.

For all the scenarios, the urban demand can be taken directly from the Dutch One Health SSPs^2^. These also provide us with total ‘rural’ land use (i.e. pasture and crops) and total ‘nature’ land use (i.e. forest and non-forest nature). Here ‘nature’ was defined as any land type which was neither urban nor agricultural. It therefore only remains to determine how these rural and nature categories should be divided. To do this we used information from the Dutch One Health SSPs, as well as other existing scenarios, national and international commitments and various literature sources. The scenarios used considered the effects of changes in climate, economics and other relevant factors, making them an invaluable source of information. Our demands for each SSP are shown in Table 2 and an explanation of our choices is given below.

**Table 2:** Demand requirements for each SSP scenario.

	Urban	Pasture	Crops	Forest	Non-forest nature
2018	538,000	1,055,800	1,456,000	302,400	196,800
SSP1	603,300	750,000	1,432,600	402,400	360,700
SSP3	532,400	891,800	1,415,000	327,400	382,400
SSP4	603,300	770,000	1,448,100	336,900	390,700
SSP5	745,300	911,500	1,359,900	311,900	220,400

All values are given in hectares rounded to the nearest 100, since this is the unit used by DynaCLUE.

SSP1 (Together Green): From the Dutch One Health SSP1, we know that urban area should cover 17% of total land area, 62% is rural and 21% is nature. It also tells us that cattle numbers are reduced by 50% by 2050 relative to 2019 and that cattle spend more time grazing and that animal welfare is improved. This suggests a large reduction in pasture area, but an increase in pasture area per cow. This is in line with the scenario ‘Natuurinclusief strikter’ from the Dutch agricultural scenarios ‘Landbouw in Nederland in 2050’^28^, which contributed to the development of this Dutch One Health SSP. This scenario describes a 31% reduction in grassland as a result of these changes in livestock numbers and management. We altered this slightly due to differences in how ‘pasture’ is categorised. Comparing this to the total ‘rural’ area, this meant that there was a small reduction in crop area compared with the present. This is in line with the global and European-level SSP1 descriptions^29,30^, as well as the Dutch One Health SSP1, which says that there is a reduction in agricultural exports, as well as increased productivity per unit area, both of which contribute to a reduction in land area. At the same time there is a growth in organic farming as well as increased investment in high-value agricultural products, such as greenhouse horticulture and fruit cultivation. It also describes an increase in agroforestry (which we class as ‘crops’). For nature, under the EU forest strategy for 2030, the Netherlands is estimated to increase forest area by around 11,500 ha by 2030^31^ and current Dutch targets are more ambitious, aiming for a 10% increase in this time period^32^. We assume these targets are easily achieved in this scenario and that considerably more land becomes forest, due to its focus on environmental sustainability. According to the ‘Groen Land’ scenario from the most recent scenarios produced by the Dutch government^33^, there will be an additional 187,000 ha of forest area by 2050, although this includes agro-forestry. It seems unlikely that this would all be fully-grown forest by 2050 and some may still count as non-forest nature for our purposes. We assumed an additional 100,000 ha of forest by 2050. This is less than in some other sustainability focused scenarios for the Netherlands^28,34^, but is still well above historic rates of forest growth for the country^34^ and thus seems like a reasonably balanced estimate. The remaining land is non-forest nature.

SSP3 (Our Town First): From the Dutch One Health SSP3, we know that urban area should cover 15% of total land area, 65% is rural and 20% is nature. It also tells us that there is a 20% reduction in cattle and a reduction in agricultural activities on poor quality land. We assume that most of the reduction in rural areas (80%) is due to reduction in pasture, with the remainder being a reduction in crop area. This is in line with the European-level SSP3 description^30^. For nature, only a small amount of new nature is purposefully created, but there is a large increase in unmanaged land, which counts as nature for our purposes. Some abandoned land and existing non-forest nature will eventually succeed to forest, but this takes several decades (estimates range between 20 and 130 years), depending on species and local conditions^35–39^. In the Netherlands, one study found that it took at least 40 years for pines to become the dominant species on inland sand dunes^40^. Since we are only considering a 32-year time period (2018–2050), it seems unlikely that many areas will succeed into forest. Summing the total area which is lost from urban and rural land uses with the existing non-forest nature area in 2018, and assuming that abandonment occurs at a linear rate and that succession to forest takes 30 years (this is a low estimate, but some nature areas may already be part-way there and we wanted to ensure that at least some succession happened in the time period), this gives us around 25,000 ha of forest gained over the time period. The remaining land is non-forest nature.

SSP4 (The Green Gulf): From the Dutch One Health SSP4, we know that urban area should cover 17% of total land area, 63% is rural and 20% is nature. It also tells us that there is a 50% drop in cattle numbers, that they spend less time grazing, that there is a reduction in major agricultural activities on poor quality land and that there are more small-scale organic farms. This suggests that most of the drop in ‘rural land’ will come from pasture and that there will be just a small reduction in crop area. For nature, we assume that the target to increase forest areas by 11,500 ha by 2030 under the EU forest strategy is met^31^, since the EU is a powerful force in this scenario and there is an emphasis on reducing emissions^2^. However, their lack of care for the natural environment suggests that there will be little intentional forest growth beyond this. There is however a great deal of unmanaged nature and some of this will naturally turn to forest. Performing similar calculations as for SSP3 and assuming that 87% of abandoned land and non-forest nature has the potential for succession (this excludes the wealthy areas and areas near centres for knowledge and business, which are well-maintained in this scenario, see materials on Dryad^41^ for an explanation of how this was calculated), this gives us around 23,000 ha of forest gained via succession over the time period, totalling an extra 34,500 ha overall. The remaining land is non-forest nature.

SSP5 (After us comes the Deluge): From the Dutch One Health SSP5, we know that urban area should cover 21% of total land area, 64% is rural and 15% is nature. It also tells us that there is a 10% reduction in cattle, a reduction in animal welfare and that there are reductions in both arable farmland and intensive grassland. We allocated 40% of the reduction in ‘rural land’ to crops and 60% to pasture. This increases the number of cows per hectare (representing the reduction in animal welfare) and is in line with the European-level SSP5 description^30^. For nature, there is only a very small increase in this scenario. The purpose of nature is recreation rather than environmental protection and nature areas become more park-like, suggesting little incentive to increase forest areas. Nature areas are not well-managed outside populated areas, suggesting that some may naturally become more forested. Performing similar calculations as for SSP3 and assuming that 67% of non-forest nature has the potential for succession (excluding areas near population centres, see materials on Dryad^41^ for an explanation of how this was calculated), this gives us around 9,500 ha of forest gained via succession over the time period. The remaining land is non-forest nature.

### Determine possible predictors of change (from literature) and use logistic regression to determine how they affect land use type (‘Location characteristics’)

From looking at other land use change studies^42,43^ and discussions with experts in land use modelling, we derived a list of predictors which could influence land use type and found spatial data for each of these predictors. All data were transformed to a 1 km grid and we removed those predictors which were highly correlated with others (>0.7 or <−0.7, see accompanying materials on Dryad^41^). Those remaining are shown in Table 3.

**Table 3:** Possible predictors affecting land use type and data sources.

Predictor	Source
Altitude	*Actueel Hoogtebestand Nederland 3 DTM, 2023* [Dutch Current Elevation] (https://www.nationaalgeoregister.nl/geonetwork/srv/dut/catalog.search#/metadata/b0c89cbf-8f30-414b-b6d3-cad222869f6c)^64^
Soil bulk density	European Soil Data Centre: Topsoil physical properties for Europe, 2015 (https://esdac.jrc.ec.europa.eu/content/topsoil-physical-properties-europe-based-lucas-topsoil-data)^65^
Soil clay content	European Soil Data Centre: Topsoil physical properties for Europe, 2015 (https://esdac.jrc.ec.europa.eu/content/topsoil-physical-properties-europe-based-lucas-topsoil-data)^65^
Soil pH	European Soil Data Centre: Maps of soil chemical properties at European scale based on LUCAS 2009/2012 topsoil data: pH in CaCl2 solution, 2019 (https://esdac.jrc.ec.europa.eu/content/chemical-properties-european-scale-based-lucas-topsoil-data)^66^
Groundwater level	Klimaateffectatlas: Impacts > Mean lowest groundwater level, current scenario, based on National Water Model 2019 (https://www.klimaateffectatlas.nl/en/)^44^
Salinity	Chloride concentration in surface water. *Nederlands Hydrologisch Instrumentarium: Actualisatie zoutmodellering NHI (oppervlaktewater)* [Dutch Hydrological Instrumentarium: Update salt modeling NHI (surface water)], 2021^67^
Flood risk	Klimaateffectatlas: Impacts > Flood depth at extremely low, low, medium and high probabilities, based on National Water and Flood Information System (LIWO)/Floods Directive (ROR), 2021 (https://www.klimaateffectatlas.nl/en/)^44^. We used 0.1*(max depth of ‘1 in 10 year’ flood event) + 0.01*(max depth of ‘1 in 100 year’ flood event) + 0.001*(max depth of ‘1 in 1000 year’ flood event) + 0.00001*(max depth of ‘1 in 100,000 year’ flood event)
Agricultural land price	Silvis, H & Voskuilen, M., average values for 2017–2020^68^
Distance to nearest airport	Major international airports. World Bank: Global Airports: Airports geospatial data, 2020^69^
Distance to nearest major city	Cities with more than 150,000 people. *Centraal Bureau voor de Statistiek: Bevolking op 1 januari en gemiddeld; geslacht, leeftijd en regio* [Statistics Netherlands: Population on January 1 and average; gender, age and region], data from 2019^70^
Distance to nearest motorway	A-roads. Rijkswaterstaat: NWB-wegen, data from 01/01/2019^71^
Distance to nearest port	World Port Index: UpdatedPub150.csv, downloaded 30/01/2023^72^
Distance to nearest train station	ProRail: Spoorwegen > WFS file > stations, selected stations open in 2019^56^
Distance to nearest major waterway	*Nationaal Wegen Bestand – Vaarwegen* [National Route Database – Waterways], 2023^73^
Average annual temperature	Average 2000 – 2019. KNMI (Royal Netherlands Meteorological Institute): Gridded daily mean temperature in the Netherlands^57^
Average total precipitation	Average 2000 – 2019. KNMI (Royal Netherlands Meteorological Institute): Gridded daily precipitation sum in the Netherlands^58^
Distance to nearest urban/pasture/crops/forest/non-forest nature area	CORINE2018^23^

Other predictors which were considered were income and population. While these are very good predictors of current land use, they bring up questions of direction of causation, i.e. do people cause urbanisation or does urbanisation attract people? Also, including these would mean we would have to create spatial maps of future income and population for each of our scenarios in order to generate future land use maps. This seemed too much like us deciding on future land use rather than letting the model tell us, and so these predictors were omitted. There are also potential feedback effects for some of the predictors we have included. For example, flood risk affects land use, but is also affected by it. We did not account for these feedback effects in our modelling process, due to the complexities involved.

To determine the effect of each of the predictors on land use type, we performed logistic stepwise bi-directional regression based on the Akaike information criterion (AIC), using data on 2018 land use. Only predictors which were deemed relevant for each land use type were included (e.g. ‘Distance to airport’ was not included for forests). The AUC-ROCs (area under the curve of the receiver operator characteristic) are shown in Table 4 and full results can be found in the in the accompanying materials on Dryad^41^. The regression coefficients are used directly as inputs to DynaCLUE. Of course this represents a simplified situation. In reality, many of the predictors are themselves influenced by land use type and so cannot technically be said to be true ‘predictors’. However, they still provide valuable information on local suitability for certain land use types.

**Table 4:** AUC-ROCs for the logistic regression outcome for each land use type.

Land use type	AUC
Urban	0.995
Pasture	0.998
Crops	0.998
Forest	0.996
Non-forest nature	0.986

### Determine values for predictors which are not constant over time (‘dynamic predictors’)

For each predictor, with the exception of those for ‘distance to airport/motorway/station’, we generated a map for 2050, then linearly scaled it from the original 2018 map to produce a map for each year of the simulation. Several predictors are based on previous Dutch climate scenarios. A summary of the scenarios used for each predictor is shown in table SM2 in the supplementary materials on Zenodo^22^. The methodology used for each dynamic predictor is detailed below:

#### Altitude

We used predictions for soil subsidence for 2050 and subtracted this from the current altitude [Klimaateffectatlas: Impacts > Soil subsidence 2020–2050 (https://www.klimaateffectatlas.nl/en/)]^44^.

#### Groundwater

We used predictions for average lowest groundwater level for 2050. These were produced by the Dutch National Water Model^45^ and accounted for climatic, socio-economic and environmental changes using the same scenarios as were used to develop the Dutch One Health SSPs, so the underlying assumptions are the same [Klimaateffectatlas: Impacts > Mean lowest groundwater level (https://www.klimaateffectatlas.nl/en/)]^44^.

#### Salinity

Future salinity data for 2050 came from a single ‘high temperature’ scenario^46^, which was a precursor to other high temperature scenarios used in this study. We used this as our 2050 salinity map for SSPs 3 and 5. SSPs 1 and 4 required a less extreme salinity map. After scaling the map for SSPs 3 and 5 to give us a map for each year of the simulation, we chose the map from three quarters of the way through the time period (2042) to act as our 2050 map for SSPs 1 and 4. This was because in more recent Dutch future climate scenarios^47^, low temperature scenarios are predicted to have around three quarters the sea-level rise of high temperature scenarios in 2050.

#### Flood risk

For the ‘present day’ flood risk, data was available for the maximum depth of a ‘1 in X year’ flood event, for different values of X [Klimaateffectatlas: Impacts > Flood depth at extremely low, low, medium and high probabilities, 2021 (https://www.klimaateffectatlas.nl/en/)]^44^. For future flood risk, the available estimates gave the approximate probability of certain flood depths being reached [Klimaateffectatlas: Impacts > Location-specific probability of flooding for 0, 20, 50 and 200 cm (https://www.klimaateffectatlas.nl/en/)]^44^. It was possible to convert between these two measures, but this required certain assumptions to be made, since for the future estimates the depths and probabilities were given as ranges rather than particular values. Only one future scenario was available in the Klimaateffectatlas, so by varying the values chosen within each range, we produced different future flood risk maps for each of our SSP scenarios. The precise values used were chosen to produce sensible predictions relative to the present day flood risk (i.e. not orders of magnitude dissimilar) and are shown in table SM3 in the supplementary materials on Zenodo^22^. The considerations for each scenario were taken from the Dutch One Health SSP descriptions^2^ and are as follows:

SSP1: Good flood defences, less sea-level rise and fewer and less intense extreme weather events than other scenarios.

SSP3: Lack of new flood defences, and existing defences not well maintained, more frequent and intense extreme weather events.

SSP4: Good flood defences in some areas, but not in others. Less sea-level rise and fewer and less intense extreme weather events than SSPs 3 or 5, but not as good as in SSP1.

SSP5: Good flood defences, but higher sea-level rise and more frequent and intense extreme weather events.

It should be noted the future flood risk maps used did not account for all factors which might affect flood risk. For example, they did not consider the effect of land use change, which could have a major effect on flood risk, especially in the event of land cover becoming much more impervious (i.e. with major shifts towards urban areas). It is therefore possible that future flood risk could be greater than what was used here, especially under high urbanisation scenarios such as SSP5. On the other hand, the quality of flood defences will also come into play here. An increase in impervious area coupled with higher and well-maintained flood defences may see little increase in flood risk.

#### Agricultural land price

Land price depends on a huge range of factors. To estimate future land price we performed linear regression, based on current land prices. The covariates used were: altitude, soil bulk density, soil clay content, soil pH, groundwater level, salinity, flood risk, distance to nearest motorway, distance to nearest port, distance to nearest major waterway, average annual temperature, average total precipitation and proportion of protected nature area within a 50 km radius. All data was the same as that used for the land use modelling. All these covariates were highly significant and the model had an R^2^ value of 0.49. Full results are available on Dryad^41^. This model was used to predict future land price for each SSP, applying the same covariate inputs as were used to predict future land use. The land price predictions were scaled relative to current prices, so we only consider relative spatial differences, rather than changes in absolute prices.

#### Distance to nearest airport

For SSPs 1 and 4, we kept the number and locations of airports constant. There are plans to open an existing airport in Lelystad to commercial holiday flights (it currently only operates private flights)^48^, but there is considerable debate about whether this should go ahead. Since these two scenarios prioritise low emissions it seemed unlikely that a new commercial international airport would be welcome in this scenario. In SSP3 we determined that the airport in Lelystad would open soon (2025), since the main hold-ups are environmental concerns, which would carry little influence under this scenario. However, the poor economy and reduced trade and travel would have significant impacts on airport profitability. We determined that it was unlikely that the new airport could remain open for long under such conditions and so we assumed it would close around 2035. The economic situation in this scenario would also likely affect other airports. To reflect this, we assumed that Eelde airport near Groningen would also close (we chose 2040 for this), since it is in an area which is expected to suffer considerable population loss. In SSP5 we also assumed that the Lelystad airport would open, due to lack of environmental concerns under this scenario. However, here economic growth is strong and trade and travel are both high, so the airport was assumed to remain open.

#### Distance to nearest motorway

We added new motorway segments which have already been completed since 2018. Currently, motorway developments in the Netherlands focus on renovation and widening rather than the creation of new motorways^49^. The three planned extensions are shown in table SM4 in the supplementary materials on Zenodo^22^. Scenario-specific considerations were as follows:

SSP1: Reduced travel and high environmental awareness led us to assume no new motorway construction beyond those already planned.

SSP3: There is reduced travel, as well as a lack of investment or inter-regional cooperation, making it unlikely that there would be new motorways built. We assumed the first and second planned developments would take place under SSP3, but not the third, since this in a much earlier stage and more likely to be abandoned given a lack of investment or cooperation.

SSP4: There are conflicting forces at play in this scenario: the priority on emissions reduction means that there would be little incentive to build new motorways, especially since many people still use fossil-fuel powered vehicles. On the other hand, people travel a lot in this scenario, and increased congestion is likely to lead to more demand for new motorways (or at least the expansion of existing motorways). There is a focus on supporting the elite social class, as well as maintaining a good environment for business. As such they will want business and knowledge centres to be well connected. We assumed that all three planned developments would take place, as well as adding small sections around the cities of Haarlem, Den Haag and Eindhoven to increase their connectedness to the motorway network. These additions were taken from the ‘Ruimtelijke verkenning 2023’ scenarios^50^ and involved upgrading N-roads to A-roads.

SSP5: This scenario involves large increases in both population and travel, indicating a need for many new motorways. There is also weak environmental regulation and little concern for the environment, suggesting that there would be few barriers to road construction. We assumed all the planned developments would take place, as well as the additions included in SSP4. In addition, we included all motorways shown in the ‘Ruimtelijke verkenning 2023’ scenarios^50^. These were mainly upgrading N roads to A roads, but there were also a small number of completely new road sections. The new motorways were mainly in the provinces of Zeeland, Flevoland, Friesland, Groningen, Drenthe, Overijssel and Gelderland.

#### Distance to nearest station

There are several railway stations which are planned to open in the coming years, as shown in table SM5 in the supplementary materials on Zenodo^22^. Scenario-specific considerations were as follows:

SSP1: Public transport is popular in this scenario and receives a lot of investment. We assumed that all planned stations would go ahead, and that 15 additional stations would also be created by 2050. These were selected from stations which have previously been proposed^51,52^, choosing towns which do not currently have a station but are close to a trainline (or which would require only a short extension of an existing trainline) and are relatively far from other stations.

SSP3: In this scenario the population is shrinking, the economy is in a bad state, there is little investment and little concern for mitigating climate change. People also travel less than at present. With this in mind, it was decided that it was likely that stations would close in this scenario, particularly in rural areas. In 2035 we closed the 3 least busy stations in the country^53^, then in 2035 the next 5 least busy, and in 2045 the next 10 least busy stations. We also assumed that none of the planned stations would go ahead.

SSP4: This is a very unequal scenario. There is high investment in the prosperous areas of the Randstad, but not in more peripheral areas. While there is an emphasis on reducing emissions, which should encourage promotion of public transport, there is little appetite for providing resources to small communities in rural areas. We decided that the planned new stations in South Holland would go ahead, but not the others. Similarly, those stations which were added in SSP1 which are situated in North and South Holland were added, but not the others. In contrast, little used stations in rural areas were closed. In 2035 we closed the 5 least busy stations in the country^53^, and in 2045 the next 5 least busy stations.

SSP5: This is a high investment scenario with sprawled urban development. While they have little interest in reducing emissions, there is still an emphasis on looking after people and providing necessary resources. We therefore added all the same new stations as in SSP1, with the addition of a station at Lelystad Airport.

#### Temperature and precipitation

The temperature input for our model is ‘average daily mean temperature over the previous 20 years’, while for precipitation it is ‘average annual rainfall over the previous 20 years’. Daily raster files were available for temperature and precipitation for different emissions scenarios for the years 2035 to 2050 [KNMI’23 scenarios: tas_Xx_2050_interp.nc and pr_Xx_2050_interp.nc files, averaging the n and d (wet and dry) variants]^54^. While this does not cover the whole twenty year period prior to 2050, we decided that it was close enough and we used these files to calculate average daily mean temperature and average annual rainfall for each scenario. We used a low emissions scenario for SSP1, medium emissions for SSP4 and high emissions for SSPs 3 and 5.

### Define spatial restrictions and preferences affecting land use change under each scenario (‘Spatial policies and restrictions’)

DynaCLUE allows us to define gridsquares where land use does not change. It also allows us to increase the probability of certain land use types in specified cells (termed ‘location specific preference addition’). The Dutch One Health SSP descriptions^2^ provide certain spatial information which can be translated into spatial restrictions and preferences. We applied the measures shown in table SM6 in the supplementary materials on Zenodo^22^.

### Define additional model parameters and inputs

The main inputs for DynaCLUE were determined in steps (2) to (6), but the model also requires several additional parameters. All inputs used can be found on Dryad^41^, but the most important additional parameters are explained in more detail below.

- Land use history: Land use history was derived from the CORINE land cover dataset^55^.
- Dynamic predictors: Many predictors we used remain relatively constant over time, at least over the time scale we are considering, up to 2050. However, some change and must be updated for each year simulated. The dynamic predictors used were altitude, groundwater level, salinity, flood risk, agricultural land price, distance to nearest airport, distance to nearest motorway, distance to nearest train station, temperature and precipitation. Details of how these were determined are in the accompanying materials on Zenodo^22^.
- Parameters relating to convergence criterion and the weights to assign to the location specific preference additions: This was a matter of finding values which allowed the model to converge while producing maps in line with the SSP descriptions. This was done by trial and error.

### Use (2) to (7) as inputs for DynaCLUE and run the model to create land use maps for each scenario

**Fig. 2:**
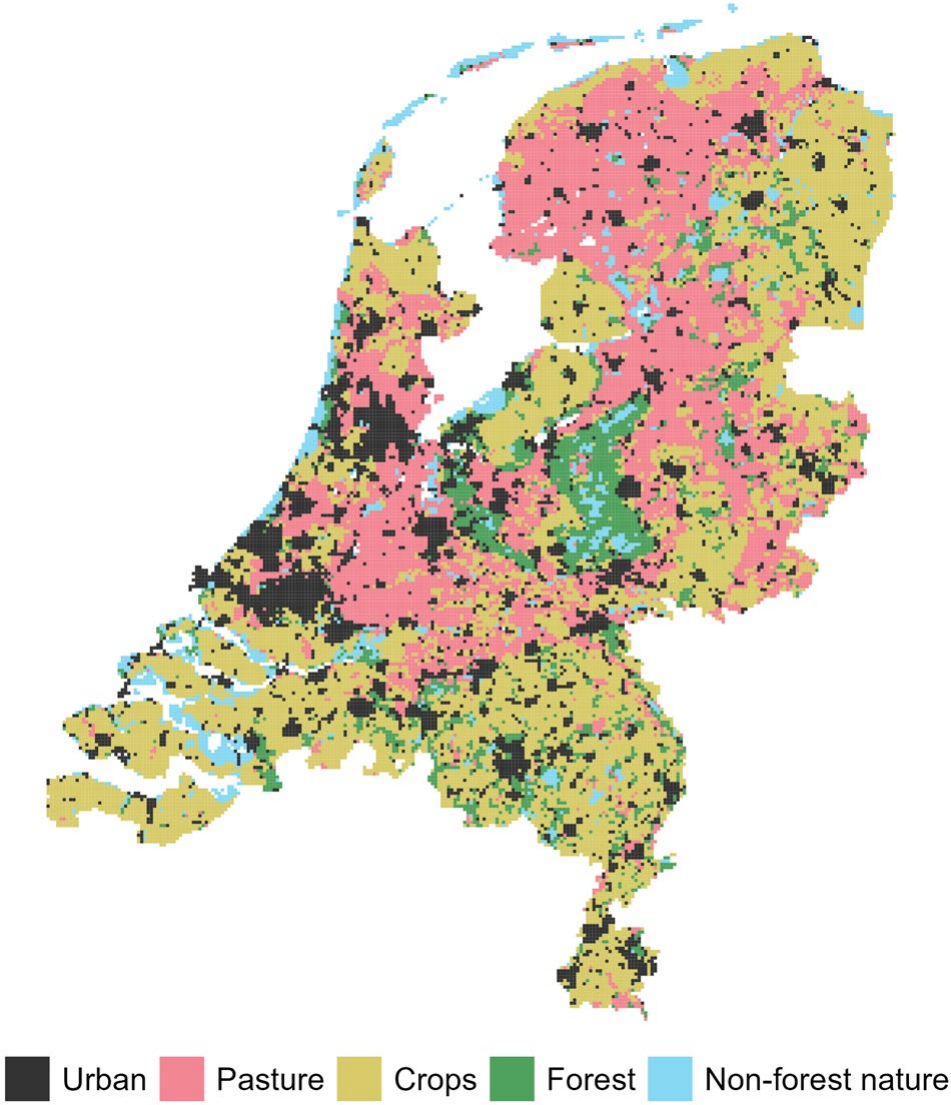
Current land use in the Netherlands, based on the CORINE 2018 land use^23^.

**Fig. 3:**
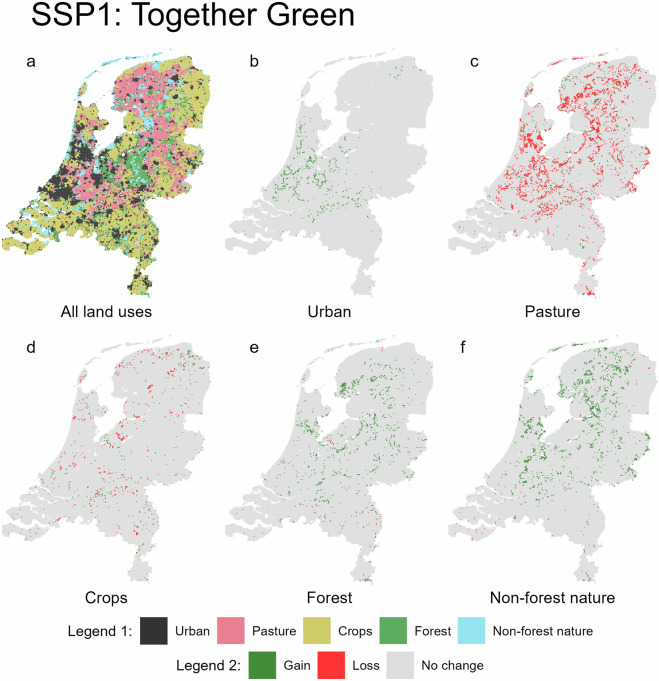
Predicted land use for 2050 under the SSP1 scenario (**a**, legend 1), with accompanying maps showing gains and losses for each land use type (**b**–**f**, legend 2).

**Fig. 4:**
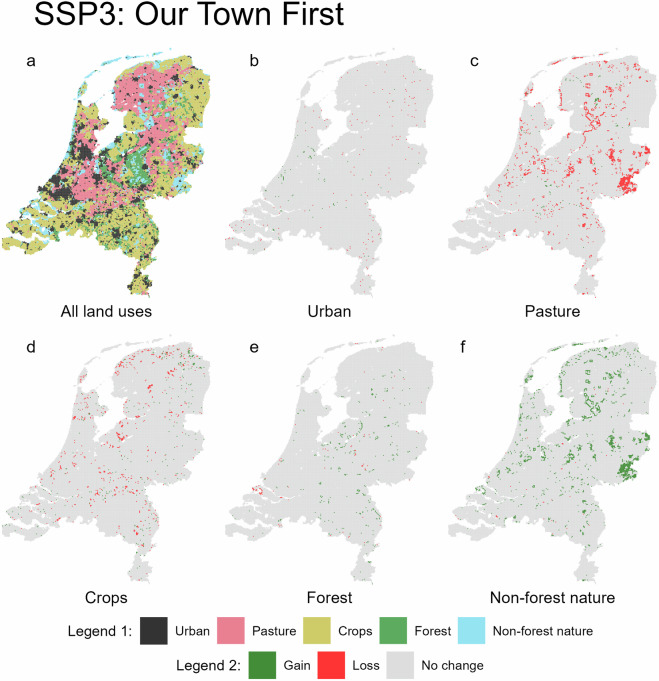
Predicted land use for 2050 under the SSP3 scenario (**a**, legend 1), with accompanying maps showing gains and losses for each land use type (**b**–**f**, legend 2).

**Fig. 5:**
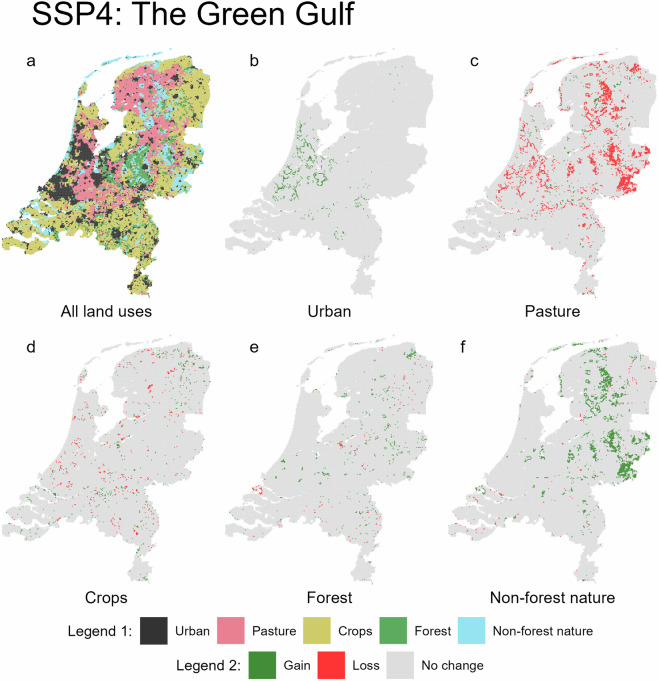
Predicted land use for 2050 under the SSP4 scenario (**a**, legend 1), with accompanying maps showing gains and losses for each land use type (**b**–**f**, legend 2).

**Fig. 6:**
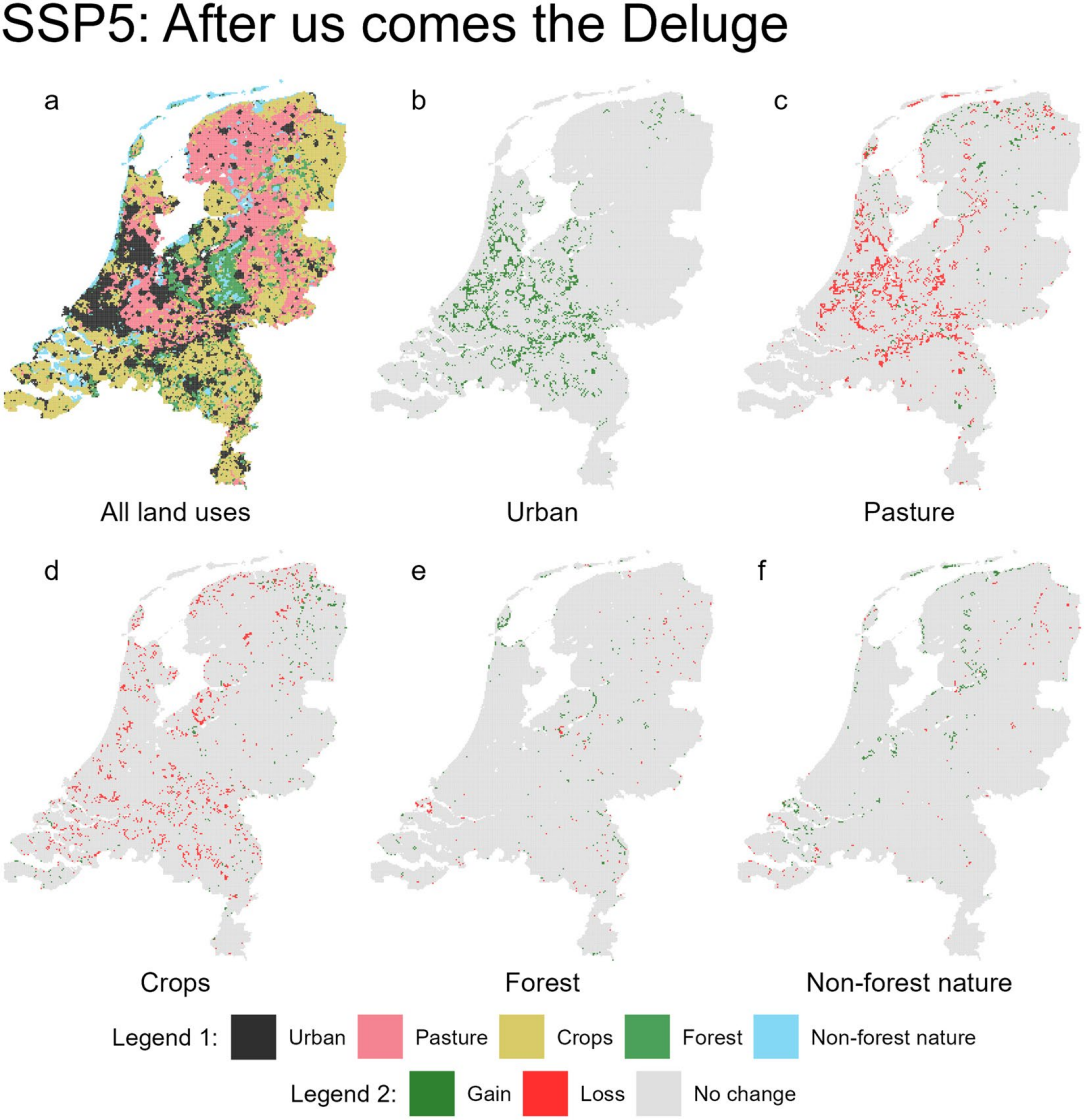
Predicted land use for 2050 under the SSP5 scenario (**a**, legend 1), with accompanying maps showing gains and losses for each land use type (**b**–**f**, legend 2).

The current (2018) land use map is shown in Fig. 2. The final land use maps and their changes from the current land use are shown in Figs. 3–6.

Together with the qualitative aspects of the Dutch One Health SSPs^2^, these land use maps will enable detailed modelling of a wide variety of future health challenges in the Netherlands. For example, the distribution and abundance of certain disease vectors is known to be dependent on land use; these maps will enable detailed modelling future vector-borne disease risk. Alternatively, they could also be used to estimate future groundwater quality, air pollution, or a whole range of other health issues. We hope that these land use maps will prove a valuable tool for future health research in the Netherlands, and that the methodology and assumptions used will inform research in other urban deltas.

## Data Records

The future land use maps for each scenario are available as raster (.asc) files. Land use types are referred to in these files by numbers: 0 - Urban, 1 - Pasture, 2 - Crops, 3 - Forest, 5 - Non-forest nature. To enable users to see changes under each scenario, the 2018 land use is also available as a raster file: cov_all.0 (this is the filename used by DynaCLUE for the starting land use).

All model inputs and model outputs are available on Dryad^41^.

All raster files are on a 1 km grid with CRS EPSG: 28992.

In addition, several supplementary tables are available on Zenodo^22^.

The available files are shown in Tables 5–7.

**Table 5:** Data files available on Dryad^41^: DynaCLUE inputs for each scenario and for validation.

File name	File type	Description	Relevant section in this paper
age.0 (validation only)	raster	Length of time each grid cell has held its current land use type	Technical validation
alloc1.reg	text	Regression coefficients for each land use type	Methods (4)
allow.txt	text	Describes which land use changes are permissible	Methods (2)
cov_all.0	raster	Starting land use	
demand.in1	text	Demand for each land use type per year simulated	Methods (3)
locspec < X > .fil	raster	Describes areas with increased probability for the given land use type (location specific preference addition). X is the land use type (0: Urban, 1: Pasture, 2: Crops, 3: Forest, 5: Non-forest nature)	Methods (6)
main.1	text	Lists model parameters	Methods (7)
region_ < X > .fil	raster	Describes areas where no land use change is possible. X is the scenario or validation	Methods (6)
sc1gr < X > . < Y>	raster	Files for each predictor (altitude, flood risk, etc.). X is the predictor number (see predictor_names.txt in *Other*). Y is either ‘fil’ or a number indicating the year of the simulation; ‘fil’ is used for constant predictors and also for the final year simulated.	Methods (4,5)

**Table 6:** Data files available on Dryad^41^: Results.

File name	File type	Description	Relevant section in this paper
SSP1.asc, SSP3.asc, SSP4.asc, SSP5.asc	raster	Final land use maps for each scenario	Methods (8)
Validation_CORINE.asc	raster	Used for validation: CORINE 2018 land use	Technical validation
validation_prediction.asc	raster	Output of validation: model prediction of 2018 land use	Technical validation

**Table 7:** Data files available on Dryad^41^: Other.

File name	File type	Description	Relevant section in this paper
correlations.xlsx	spreadsheet	Correlations between possible predictors	Methods (4)
currentLU.asc	raster	Current (2018) land use in the Netherlands, using our 5 land use types	Methods (1)
Land_types.txt	text	Lists how the CORINE land use types were classified into our 5 land use types.	Methods (1)
LandPrice_RegressionOutput.csv	csv	Output of regression analysis used to enable the prediction of future agricultural land prices	Methods (5)
predictor_names.txt	text	Lists the number used for each predictor	Methods (4,5)
predictorFiles.zip	zip	Includes all the predictor data used in the logistic regression analysis	Methods (4)
Predictors.txt	text	List of predictors used for each land use type for the logistic regression analysis	Methods (4)
rasterTemplate.asc	raster	Blank raster, providing the grid which is used in all the analysis	All
regressionOutput.csv	csv	Output of logistic regression analysis	Methods (4)
SSP4demand.tif, SSP5demand.tif	raster	Used for calculating potential forest succession as part of estimating future demand	Methods (3)

## Technical Validation

We validated our model by testing how well it predicted historic land use changes. We used 1990 as the start year, using the CORINE land cover map from this year^24^. Then we ran it for 28 years and compared the results with the 2018 CORINE land cover map^23^. To perform this validation, we needed to create appropriate model inputs, just as for making future predictions. These were as follows:

*Land use type specific conversion settings*: The same as for future predictions.

*Land use requirements (demand)*: Final demand was taken from the 2018 CORINE land cover map^23^.

*Location characteristics*: The same as for future predictions.

*Spatial policies and restrictions*: Protected nature areas could not change land use type. Some gridcells in these areas were not currently classified as nature. For these we allowed them to change but set a preference for them to change to either forest or non-forest nature

*Land use history*: Randomly generated with a maximum of 30 years with no change in land use.

*Dynamic predictors*: These were agricultural land price, distance to train station^56^, temperature^57^ and precipitation^58^. Details on the calculation of these predictors are shown in Table 8. Others could not be included due to limitations on data availability.

**Table 8:** Derivation of dynamic predictors for model validation.

Predictor	Source	Derivation
Agricultural land price	Silvis, H & Voskuilen, M., average values for 2017–2020^68^	Prices from 1990, 2000 and 2013 were scaled so that the highest values were equal to the highest value from the 2017–2020 average (this removes inflation, but it is relative spatial differences rather than absolute prices that matter). We then linearly interpolated between the different time points to get values for each year.
	Luijt, J & Voskuilen, M., values for 2013^74^	
	*Centraal Bureau voor de Statistiek: Landbouwgrond; koop - en pachtprijzen, regio, 1990–2001* [Farmland; purchase and lease prices, region, 1990–2001], yearly values per province, averaging prices for leased and unleased land, data from 1990 and 2000^75^	
Distance to train station	ProRail: Spoorwegen > WFS file > stations, selected stations open in 2019^56^	The data included details of when each train station opened, so we could calculate the distance to the nearest train station for each year.
Temperature	KNMI (Royal Netherlands Meteorological Institute): Gridded daily mean temperature in the Netherlands^57^	Calculated a 20-year moving average to get values for each year.
Precipitation	KNMI (Royal Netherlands Meteorological Institute): Gridded daily precipitation sum in the Netherlands^58^	Calculated a 20-year moving average to get values for each year.

**Fig. 7:**
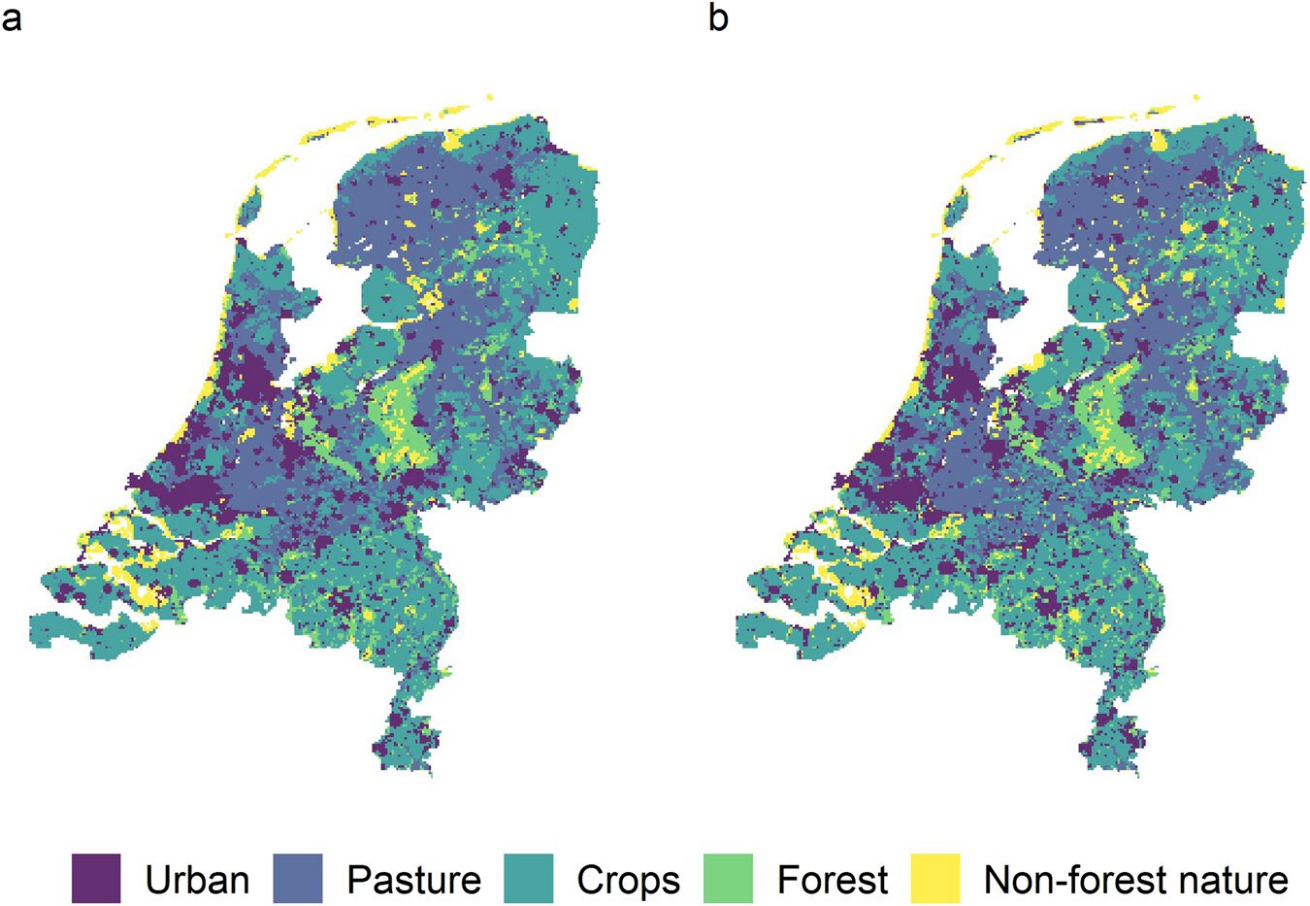
Predicted (**a**) and reference (**b**) land use for the year 2018.

The model prediction and the CORINE 2018 land use maps are shown in Fig. 7.

Out of many different possible measures of model quality, we used several different metrics to get an overview of how the model was performing in a range of respects. A confusion matrix was generated, using the R caret package^59^. Overall accuracy (the percentage of gridcells which were accurately predicted) was calculated for both the original resolution and at coarser resolutions, to remove the effect of near-misses and to show how well the general pattern matched^60^. To see how the model performed for different land use classes, we calculated producer’s and user’s accuracies for each land use type. Producer’s accuracy is the number of correctly classified cells of a certain type, divided by the total number of cells of that type in the reference dataset (in this case, CORINE 2018). User’s accuracy is the number of correctly classified cells of a certain type, divided by the total number of cells of that type predicted by the model^61^. Of course, many gridsquares remained unchanged over this time period. To ensure that our model was indeed an improvement over just keeping everything constant, we also looked at how well the 1990 map performed as a predictor of 2018 land use. This gave us a null model against which to evaluate the performance of our model.

The confusion matrix (Table 9) shows the results per land use class. The overall accuracy was 87.6% with most of the errors being between urban, pasture and crops.

**Table 9:** Confusion matrix for predicted and reference land use for 2018.

		CORINE2018				
		Urban	Pasture	Crops	Forest	Non-forest nature
Prediction	Urban	4021	738	529	46	46
	Pasture	430	9457	508	28	136
	Crops	802	128	13329	137	164
	Forest	89	70	50	2735	79
	Non-forest nature	37	164	142	80	1545

**Fig. 8:**
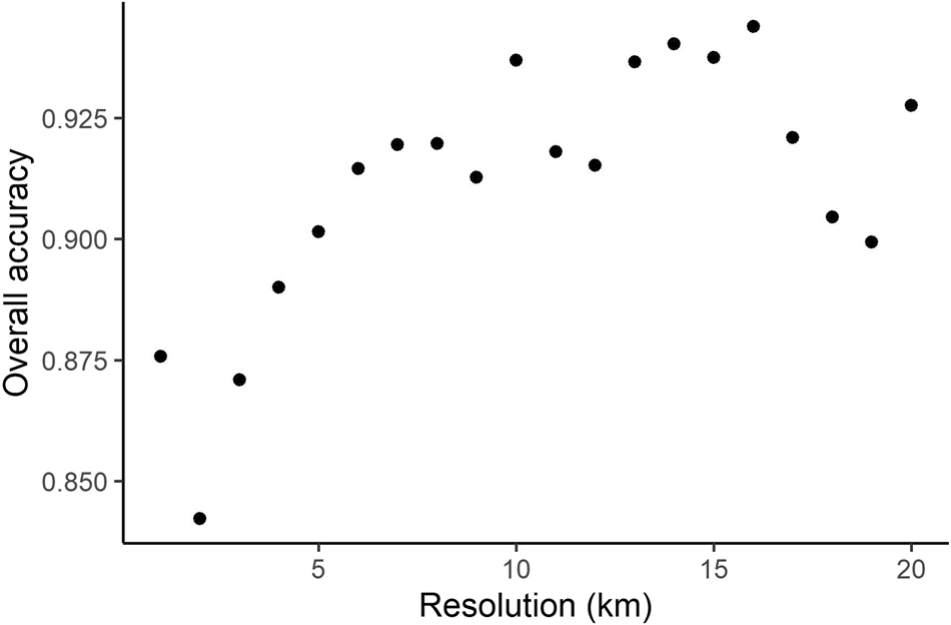
Overall accuracy of predictions compared to reference land use at different resolutions.

Comparing the accuracy at different resolutions showed that at least some of the error was due to small-scale near-misses (see Fig. 8). By looking at lower resolutions we can see that the model generally captured large-scale patterns well.

Table 10 shows the producer’s and user’s accuracies for each individual land use class at the 1 km resolution. The model was particularly good at predicting pasture, crops and forest, but less reliable for urban and non-forest nature. For future research it would be useful to consider additional predictors which are particularly relevant for these land use types. The producer’s and user’s accuracies are very similar, indicating that the model did a good job of meeting the demand requirement.

**Table 10:** User and producer accuracies for each land use class.

Land use type	User’s accuracy (%)	Producer’s accuracy (%)
Urban	74.8	74.7
Pasture	89.6	89.6
Crops	91.5	91.6
Forest	90.6	90.3
Non-forest nature	78.5	78.4

For the null model (i.e. keeping the 1990 land use constant), producer’s and user’s accuracies can be found in table SM7 in the supplementary materials on Zenodo^22^. While user’s accuracies were similar to, and in some cases better than, those shown above, the producer’s accuracies were very poor, with the maximum value being for crops at just 39.2%. This suggests that while many gridsquares were correctly classified, there were large differences in the total numbers of cells of each land use type between the 1990 and 2018 land use maps.

## Data Availability

All code was written in R v4.0.4 and we used DynaCLUE v2.0. The DynaCLUE model is available from the VU IVM Institute for Environmental Studies^62^. All code is available on Zenodo^63^. The available files are as follows: - historicChanges.R: This determines historic land use changes in the Netherlands from 1990 to 2018 (relevant to Methods (2)) - regression.R: This performs the logistic regression analysis and creates the alloc1.reg file to use as an input to DynaCLUE (relevant to Methods (4))
